# Magnetic field coupling with lunar soil simulants

**DOI:** 10.1038/s41598-023-36527-0

**Published:** 2023-06-15

**Authors:** Shanti M. Garman, Melissa C. Roth, Vincent G. Roux, Joshua R. Smith

**Affiliations:** 1 Department of Electrical and Computer Engineering, University of Washington, Seattle, 98195 USA; 2 Allen School of Computer Science and Engineering, University of Washington, Seattle, 98195 USA; 3 Off Planet Research LLC, Everett, 98201 USA

**Keywords:** Electrical and electronic engineering, Applied physics, Rings and moons

## Abstract

Wireless power transfer (WPT) using magnetically coupled resonators is being integrated into space vehicles destined for the lunar surface. The dusty soil on the Moon, called lunar regolith, is known to adhere to surfaces and is also known to contain iron, including iron oxides and metallic iron. Regolith samples are limited, and lunar soil simulants are commonly used in space science research for efforts in surface vehicle navigation, in-situ resource utilization, and power infrastructure. However, most simulants contain no metallic iron, and research involving electromagnetic field interactions with regolith would benefit from incorporating metallic iron into test samples. This work presents experimental results from tests using WPT with magnetically coupled resonators in the presence of various standard lunar simulants, plus a new iron-enriched simulant and metallic iron powders. Results for power transfer efficiency, thermal response, and frequency response are presented and demonstrate that the presence of metallic iron and its particle size are critical factors affecting the coupling of the incident magnetic field with lunar simulants and iron powder samples. The importance of particle size-to-skin depth ratio is discussed. Attenuation constants for various iron powders are estimated from experimental data and compared to those of lunar regolith and simulants.

## Introduction

Wireless power transfer (WPT) using magnetically coupled resonators (MCR) has proven to be a successful technology solution across multiple categories, ranging from low-power biomedical applications to high-power industrial applications, such as warehouse robot fleet charging scenarios^1–4^. Currently, MCR-based WPT technology is being integrated into space vehicles destined for the lunar surface, where the ability for an autonomous vehicle, such as a lunar rover, to charge wirelessly is an important component of surface power infrastructure and lunar dust mitigation strategies^5–8^. The dusty soil on the moon, called lunar regolith, is known to adhere to surfaces and is also known to contain iron in various forms, including iron oxides in the form Fe_x_O_y_ and metallic iron in the form of Fe^0^. Physical samples of lunar regolith collected by NASA astronauts during the Apollo missions are maintained by NASA under restricted access. Because access to the lunar samples is limited, the space research community has developed numerous lunar soil simulants which are readily available for space science research, especially in areas related to lunar surface infrastructure, including in-situ resource utilization (ISRU), surface vehicle mobility, power generation, power distribution, communications, construction, and dust mitigation, among others. Lunar simulants are created from Earth soils and are available with various mineralogical compositions to represent different regions of regolith known to exist on the Moon’s surface, specifically the lunar Highlands and Mare regions. Additionally, lunar simulants are available with various geophysical structures, from super-fine dust to larger rock sizes with irregular shapes, which are useful for research related to how space vehicles may interact with the regolith upon landing or while traversing the lunar surface. However, in this work, we are interested in the electromagnetic (EM) properties of lunar regolith, specifically its interactions with an incident high-frequency magnetic resonance field, namely the 6.78 MHz magnetic field designed for a deployment of a WPT system on the lunar surface. After commencing this work it was revealed that most lunar simulants do not contain metallic iron. While this is not deemed a critical parameter for research relying on simulant’s geophysical properties, it is found to be a critical parameter for research relating to EM interactions. As such, an iron-enriched simulant is introduced. In this work, we present experimental results for EM energy coupling of a high-frequency magnetic field with multiple standard lunar soil simulants, iron powders, and a customized iron-enriched lunar simulant. Our results demonstrate that particle size is a critical parameter affecting EM coupling of an incident magnetic field with particulate media such as lunar regolith. Furthermore, the particle size-to-skin depth ratio (PSSDR) of the material (also known in literature as $d/{\delta }_{S}$) is found to be a critical parameter, and its dependence on frequency is an important consideration. These particle size parameters are found to significantly impact the coupling between the field and the material, and it is concluded that in order to accurately model power dissipation and heat generation, PSSDR should be considered together with field frequency and the material’s constitutive EM parameters: electrical conductivity, complex electric permittivity, and complex magnetic permeability. Specifically, for magnetic particles with very low particle size-to-skin depth ratios (PSSDR ≪ 1), coupling is found to increase with particle size. We observe increased power dissipation, thermal response, and frequency shift with increasing particle size. Our results are directly applicable to engineers and scientists working with electromagnetic fields at interfaces with particulate media in terrestrial (Earth) or extra-terrestrial environments including ground penetrating radar (GPR), wireless networks such as cellular/LTE, WLAN, LoRa, IoT, and microwave/mm-wave power beaming networks.

The paper is structured as follows. First, theoretical background for the pursued investigation is presented, including MCR-based WPT, composition of lunar regolith and simulants, and the physical principles that govern the exchange of energy between an incident EM field and particulate materials, including the role of attenuation, skin depth, and particle size. This is followed by description of materials and methods selected for investigation, after which measured results are presented for wireless power transfer efficiency, power dissipation (heating), and frequency response of the power transfer antenna in the presence of the various lunar simulant samples and iron powders used in this study. Finally, a discussion of results and suggested areas for further research is presented.

### Theoretical background and physical principles

In this section, theoretical background is presented for wireless charging with magnetically coupled resonators in the presence of magnetic material. First, the concept of MCR-based WPT in free space is introduced. This is followed by introduction of lunar regolith and simulant material properties. Finally, the physical principles of electromagnetic coupling with materials is presented, including Joule’s Law for power dissipation, resonant frequency, field attenuation, particle size, and PSSDR.

### Wireless power transfer with magnetically coupled resonators

**Figure 1:**
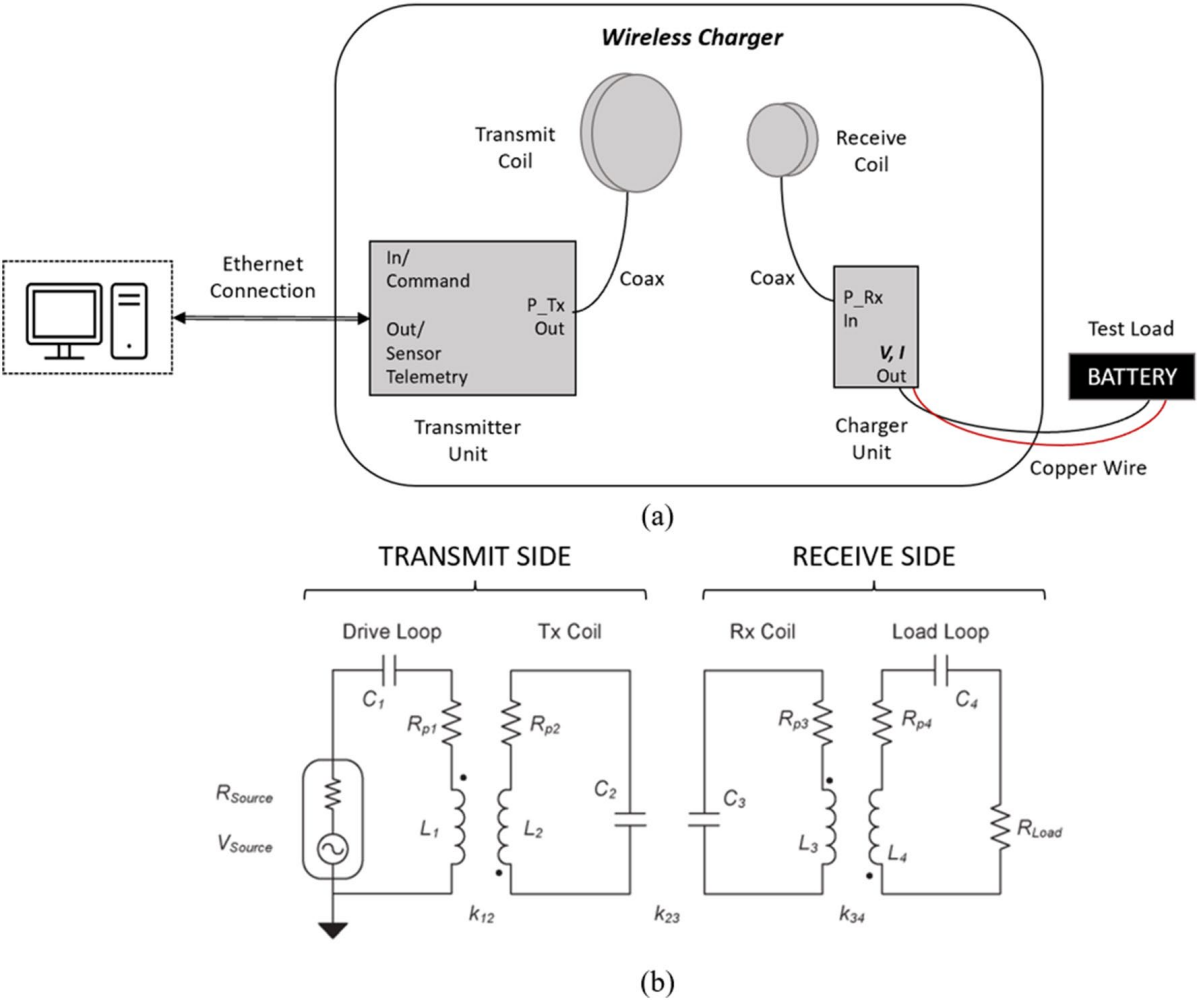
(**a**) Schematic of a typical MCR-based WPT system using a transmitter unit, charger unit, and resonant coils to charge a battery test load. (**b**) Equivalent circuit model of system. Transmit and receive side each consist of a single-turn loop plus a multi-turn loop. All elements are resonant and coupled magnetically^1^.

A typical MCR-based wireless charger topology is shown in Fig. 1. The power transfer field between the transmit coil and receive coil is near-field and non-radiative, and the magnetic flux density takes the form $B={B}_{0}cos\omega t$, i.e., a sinusoidally varying field of amplitude ${B}_{0}$ (T), angular frequency $\omega$ (rad/s), and time *t* (s)^1–4^. The circuit schematic shows lumped-element models for the resonant coils and illustrates the magnetic coupling within each coil as well as between the transmit and receive coil. Typically, coils are separated by air which has EM constitutive parameters very close to vacuum, i.e., relative magnetic permeability ${\mu }_{r}\approx 1$ (dimensionless), relative electric permittivity ${\varepsilon }_{r}\approx 1$ (dimensionless), and electrical conductivity $\sigma =0$ (S/m)^9, 10^. Hence, air has no effect on the resonance of the coils nor the coupling between the coils. However, the introduction of a material with different EM constitutive parameters, such as lunar regolith, could affect both the coil coupling and resonance.

### Lunar regolith and simulant composition

Chemical and mineralogical analysis of 79 lunar regolith samples returned during NASA’s Apollo missions show lunar regolith contains iron in the form of iron oxide (FeO) and nanophase metallic iron (Fe^0^)^11^. Reported values from these lunar soil samples show FeO content ranges from 4.2 up to 22.0 wt%, and Fe^0^ content ranges from 0.1 up to 2.0 wt%. Furthermore, it has been reported that regolith particles develop and retain an iron-rich rim about 50 nm thick which imparts a magnetic response^12^. The amount of iron in these rims varies with maturity with more mature regolith generally having more implanted iron. Terrestrial materials used for creating lunar regolith simulants do not contain Fe^0^ and often have a much lower iron content in general compared to lunar regolith. For example, lunar simulants for the lunar Highlands and Mare regions contain FeO at levels of 1.7–3.7% and 8.6–9.7%, respectively, while the amount of Fe^0^ is 0% for both types^13, 14^. Iron oxides have much lower relative magnetic permeability compared to metallic iron; relative permeability for Fe_3_O_4_ (magnetite) and Fe_2_O_3_ (hematite) is ${\mu }_{r}=$ 7 and ${\mu }_{r}= 1.00$65, respectively, compared to ${\mu }_{r}=$ 200 $\times$ 10^3^ for metallic iron Fe^0^.

### Electromagnetic coupling and material properties

In this section, physical principles are introduced for electromagnetic coupling with materials, including power dissipation, resonant frequency, field attenuation, particle size, and PSSDR.

#### Physical principles of power dissipation

When examining a material’s behavior in the presence of an electromagnetic field, it is important to consider the material’s constitutive parameters: electrical conductivity, $\sigma$ (S/m), complex electric permittivity, ${\varepsilon }^{*}$ (F/m), and complex magnetic permeability, ${\mu }^{*}$ (H/m)^9^. These parameters determine the material’s expected response in the presence of the field. For a resonant magnetic field, the dominant constitutive parameter is magnetic permeability, because this is the only constitutive parameter which is known to couple with the magnetic field; the conductivity and permittivity are known to couple with the electric field^10^. Equation (1) describes the complex magnetic permeability where ${\mu }_{r}^{*}$ (dimensionless) is the relative complex permeability of the material, and ${\mu }_{0}$ is the vacuum permeability, $4\pi \times {10}^{-7}$ H/m.

1 $${\mu }^{*}={\mu }_{r}^{*}{\mu }_{0}={\mu }^{{\prime}}-j{\mu }^{{{\prime\prime}}}$$

The real part of the permeability ${\mu }{^{\prime}}$ (H/m) indicates the material’s propensity to align its magnetization vectors, known as magnetic dipoles, with the incident magnetic field, while the imaginary part of the permeability ${\mu }^{{{\prime\prime}}}$ (H/m) indicates the amount of energy lost in the effort to rotate the magnetic dipoles following the alternating direction of the field at frequency $\omega =2\pi f$ (rad/s)^15, 16^. The relationship between dissipated power in a volume $v$, the incident field, and the material’s constitutive parameters is shown in Joule’s law,

2 $${P}_{diss}={\int }_{v}\left(\frac{1}{2}\sigma {\left|E\right|}^{2}+\frac{1}{2}\omega {(\varepsilon }^{{{\prime\prime}}}{\left|E\right|}^{2}+{\mu }^{{{\prime\prime}}}{\left|H\right|}^{2})\right)dv.$$

*E* (V/m) is electric field intensity, ${\varepsilon }^{{{\prime\prime}}}$ (F/m) is the imaginary part of the permittivity known as dielectric loss, *H* (A/m) is magnetic field intensity and is related to *B* by $B={\mu }^{*}H$. This lost power in Watts, or Joules per second, is dissipated in the form of heat through Joule heating, dielectric heating, and magnetic heating processes^10^.

#### Resonant frequency theory

As shown in Fig. 1b, the MCR-based WPT system is effectively four resonant *RLC* tanks magnetically linked by mutual inductances via coupling coefficients^1^. In such a circuit, resonance occurs when the magnetic energy stored in the inductor and the electric energy stored in the capacitor are equal, which occurs at frequency ${f}_{0}=\frac{1}{2\pi \sqrt{LC}}$ (Hz)^10^. For coupled resonators, some degree of frequency shift or antenna “detuning” can be tolerated. However, if the resonant frequency shifts outside of a given tolerance, the ability for the transmitting and receiving coils to achieve critical coupling is degraded. Accordingly, it is important to consider how materials may affect the resonant frequency of the coils, as their presence may change the effective capacitance or inductance of the circuit.

#### Physical principles of field attenuation and particle size-to-skin depth ratio

In electromagnetics, skin depth is an important parameter, as it relates to attenuation of the fields within a material. Attenuation indicates dissipation of the incident energy as described by Eq. (2). The field intensity of a plane wave traveling through material attenuates exponentially as ${e}^{-\alpha z}$ where $\alpha$ (Np/m) is the attenuation constant and *z* (m) is distance penetrated, normal to the surface. The attenuation constant itself is determined by the material’s constitutive parameters, and loss mechanisms due to magnetic loss, dielectric loss, and conductivity^9, 10^. For materials with complex magnetic permeability ${\mu }^{*}$, complex electric permittivity ${\varepsilon }^{*}$, and electrical conductivity $\sigma$, attenuation constant is calculated as:

3a $$\alpha =\omega {\left[\frac{{{\mu }{^{\prime}}{\varepsilon }{^{\prime}}-{\mu }^{{{\prime\prime}}}(\varepsilon }^{{{\prime\prime}}}+\frac{\sigma }{\omega })}{2}\left(\sqrt{1+{\left(\frac{{{\mu }{^{\prime}}({\varepsilon }^{{{\prime\prime}}}+\frac{\sigma }{\omega })+{\mu }^{{{\prime\prime}}}\varepsilon }{^{\prime}}}{{{\mu }{^{\prime}}{\varepsilon }{^{\prime}}-{\mu }{{^{\prime\prime}}}(\varepsilon }^{\prime\prime}+\frac{\sigma }{\omega })}\right)}^{2}}-1\right)\right]}^{1/2}.$$

Substituting $A={{\mu }{^{\prime}}{\varepsilon }{^{\prime}}-{\mu }^{{{\prime\prime}}}(\varepsilon }^{{{\prime\prime}}}+\frac{\sigma }{\omega })$ and $B={{\mu }{^{\prime}}{(\varepsilon }^{{{\prime\prime}}}+\frac{\sigma }{\omega })+{\mu }^{{{\prime\prime}}}\varepsilon }{^{\prime}}$, the expression can be simplified to^17^:

3b $$\alpha =\omega {\left[\frac{A}{2}\left(\sqrt{1+{\left(\frac{B}{A}\right)}^{2}}-1\right)\right]}^{1/2}.$$

Skin depth, ${\delta }_{S}$ (m), is defined as the depth at which the field attenuates to ${e}^{-1}$ or approximately 37% of its starting magnitude and is calculated by taking the inverse of the attenuation constant, ${\delta }_{S}=1/\alpha$. The skin depth of a material gives an idea of how far an incident EM field may penetrate the material and still exert EM influence on the penetrated material.

Both the characteristics of the incident field and the characteristics of the material under study are important when considering coupling mechanisms. For example, in the case of a high frequency transverse EM wave interacting with a bulk metal, both time-varying E-field and B-field are present. In this case, the material’s conductivity and complex permittivity will affect the E-field within the sample, while the complex permeability will affect the B-field. Hence, the same EM field in the presence of a nonmagnetic conductor such as copper will exhibit different behavior than in presence of a magnetic conductor such as iron. In this work, the incident field is predominately magnetic, while both magnetic and nonmagnetic materials are considered. However, the time-varying B-field induces a circulating E-field in accordance with Faraday’s law:

4 $$\overrightarrow{\nabla }\times \overrightarrow{E}=-\frac{\partial \overrightarrow{B}}{\partial t},$$

resulting in eddy currents in the conductive media, following the point form of Ohm’s law with current density, *J* (A/m^2^), written $\overrightarrow{J}=\sigma \overrightarrow{E}$. Furthermore, existing research has shown that a material’s physical structure, particularly particle size, affects the coupling^18–21^.

Regarding particle size, it has been demonstrated that PSSDR, the ratio of particle size, $d$ (m), to skin depth, is a critical parameter for EM field attenuation, or absorption, in fine metallic particles^18–21^. If $d$ is very small compared to ${\delta }_{S}$ ($PSSDR\ll 1$), an individual particle is nearly transparent to incident fields, and very little attenuation occurs as the field transits across the entire particle. However, if $d$ is very large compared to ${\delta }_{S}$ ($PSSDR\gg 1$), an individual particle mostly reflects the energy, similar to a short circuit. When the particle size is on the order of the skin depth ($PSSDR\approx 1$), peak absorption per volume occurs; a value of ~ 4.8 has been suggested as an optimal target ratio for maximum microwave absorption in nonmagnetic metal particles^20^. Furthermore, research into how coupling relates to particle size for spherical metallic particles shows that for $PSSDR\ll 1$, an individual particle heats volumetrically while for $PSSDR\gg 1$, heating occurs in a surface layer of the particle due to skin depth effects^20^. It is shown that in the limiting case for $PSSDR\ll 1$, the power absorbed by an individual spherical particle irradiated by the magnetic field component of a plane EM wave is given by:

5 $${P}_{i}=\kappa {d}^{3}{\left(\frac{d}{{\delta }_{S}}\right)}^{2},$$

where ${P}_{i}$ is energy absorbed in the individual particle in unit time (J/s or W), and $\kappa =\frac{\pi }{240}{\mu }_{0}\omega {H}^{2}$ (W/m^3^).

## Material and methods

In this section, the material and methods for the pursued investigation are presented. With consideration of the electromagnetic coupling principles presented above along with the understanding of the difference in metallic iron content between lunar regolith and standard lunar simulants, this section describes the selected lunar simulant samples and wireless charger, as well as the experimental methods employed in this investigation.

### Lunar simulant sample selection

**Figure 2:**
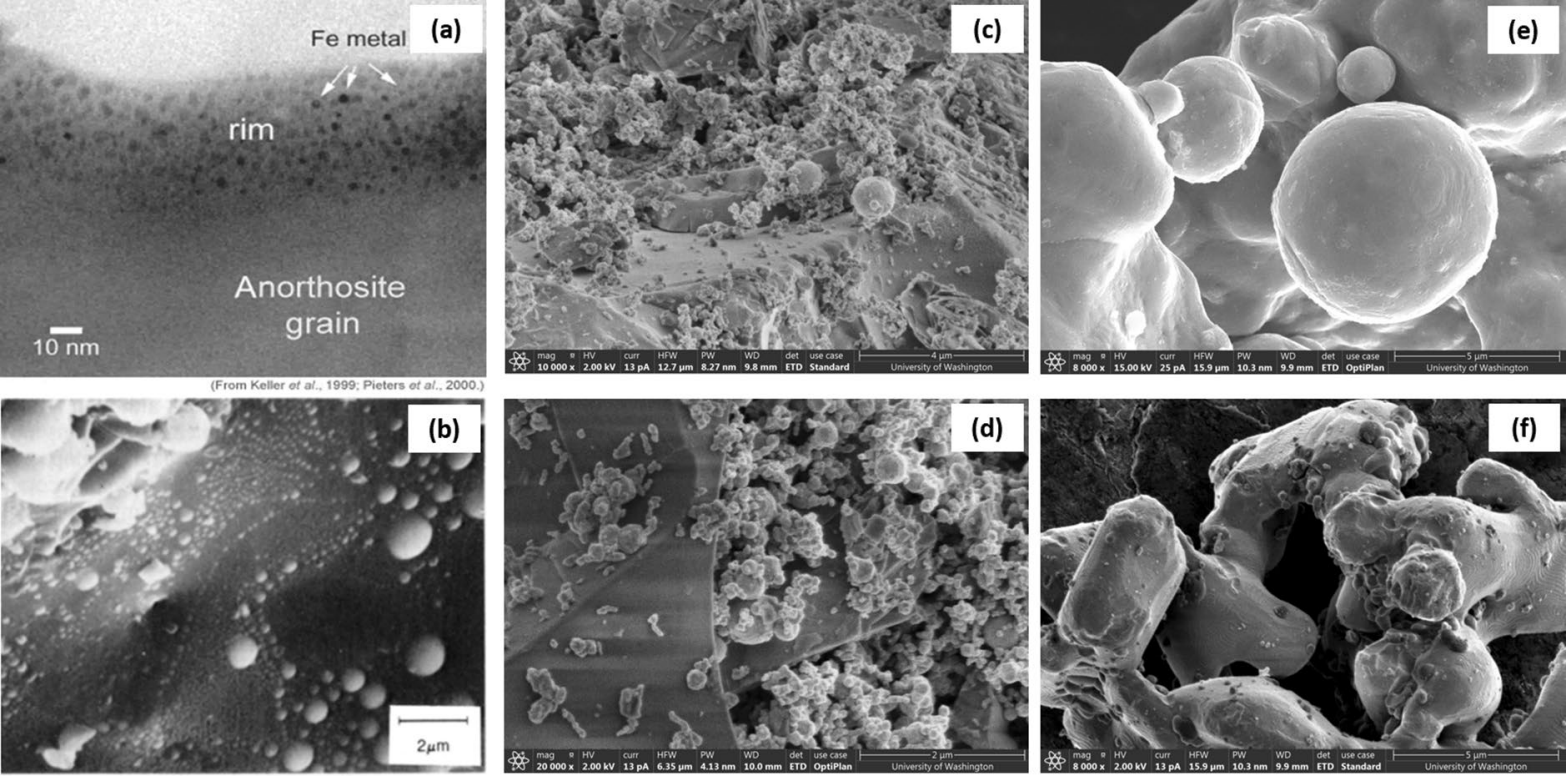
Images of actual lunar regolith samples returned from Apollo missions (**a**,**b**), manufactured lunar simulant OPRL2NS with added iron nanoparticles (**c**,**d**), and iron powders FE100 and FE325 (**e**,**f**). (**a**) Lunar regolith: Thin film section showing metallic iron (Fe^0^) at the surface of the grain^12, 22^. (**b**) Lunar regolith: Closeup SEM view of sample 10,084 showing metallic Fe mounds of 20 Å to 1 $\upmu$m diameters (NASA Photo S87-38846). It is believed that these iron particles resulted from vapor deposition during micro-meteoroid impacts^11^. (**c**) Lunar simulant: OPRL2NS, magnification: ×10,000. (**d**) Lunar simulant: OPRL2NS, magnification: ×20,000. Both images of OPRL2NS show 25 nm spherical iron nanoparticles dispersed across the surface of underlying base simulant. (**e**) Iron powder: FE325, superfine pure iron powder, particle size < 45 μm. Particles are mostly spherical. Magnification: ×8000. (**f**) Iron powder: FE100, fine pure iron powder, particle size < 150 μm. Particles are irregular, not spherical. Magnification: ×8000.

In this work, five lunar simulant samples plus six iron powder samples are selected for evaluation. Of the lunar simulants, four are standard [Lunar Highlands Simulant (LHS-1), Lunar Highlands Dust Simulant (LHS-1D), NASA Johnson Space Center lunar simulant (JSC-1A), and Off Planet Research Highlands simulant with added agglutinates (OPRH4W30)] while one is enriched with iron (OPRL2NS)^13, 14, 22^. The standard Highlands simulants are specified and provided by NASA to support the target landing location at the lunar south pole, known as the lunar Highlands. Motivation for this work to have relevance across the lunar surface leads to the selection of lunar Mare simulant JSC-1A which is created by NASA Johnson Space Center. OPRL2NS is a new iron-enriched simulant that was formulated specifically for evaluation of its effect on wireless power transfer, as it represents a regolith with a total iron content of 20.0 wt% including both the iron already present in the feedstock materials (in the form of Fe_x_O_y_) and 25 nm metallic iron particles added as an inclusion to the feedstocks. To investigate a “worst case” scenario for wireless charging in the presence of metallic iron, pure iron powders (> 98% pure metallic iron) are included in this study at two particle sizes: FE100, which has particle size of < 150 μm, and FE325, which has particle size of < 45 μm^23, 24^. Figures 2a,b show NASA’s thin section and scanning electron microscope (SEM) images which illustrate metallic iron particle size and distribution in the regolith. Figures 2c,d show SEM images of OPRL2NS where the 25 nm spherical iron nanoparticles are noticeably dispersed across the surface of underlying base simulant. Finally, Figs. 2e,f show SEM images of the iron powders, FE100 and FE325. Details about the primary chemical composition of all lunar simulants and iron powder samples used in this study are shown in Table 1. Type, mass, and density for all samples are summarized in Supplementary Table S1.

**Table 1:** Primary chemical composition of lunar simulants and iron powder samples by wt%.

Sample	Particle size	SiO_2_	Al_2_O_3_	CaO	MgO	Fe_x_O_y_	Fe^0^
LHS-1	< 400 µm	48.1	25.8	18.4	0.3	Fe_2_O_3_ 3.7	0.0
LHS-1D	< 35 µm	48.1	25.8	18.4	0.3	Fe_2_O_3_ 3.7	0.0
JSC-1A		46–49	14.5–15.5	10–11	8.5–9.5	Fe_2_O_3_ 3–4 FeO 7–7.5	0.0
OPRH4W30	< 4.75 mm	48.07	30.28	15.23	1.08	1.69	0.0
OPRL2N	< 4.75 mm	47.35	17.40	11.08	8.10	9.67	0.0
OPRL2NS	< 500 µm	41.89	15.39	9.80	7.17	8.56	11.52
FE325	< 45 µm	0.0	0.0	0.0	0.0	0.0	99
FE100	< 150 µm	0.0	0.0	0.0	0.0	0.0	98

### Wireless charger selection

A commercially available MCR-based wireless charger from WiBotic, Inc. is selected because it is specified by Astrobotic and NASA, and it serves as the technological reference for chargers which will be incorporated on lunar spacecraft under development. Specifically, the commercial system includes a TR-301 transmitter unit, 200-mm transmit coil, OC-251 charger unit, and a 100-mm receive coil^25^. The charger is configured to transmit approximately 90 W wirelessly from a 6.78 MHz transmitting unit to a receiving unit connected to a battery load.

### Experimental methods

We evaluate coupling of the magnetic power transfer field with lunar soil simulants by measuring power transfer efficiency, power dissipation, and resonant frequency shift. For each test, separation between transmit coil and receive coil is approximately 40 mm, which is the distance for maximum power transfer for the planned lunar mission. The sample is placed between the coils with both coils and the sample horizontal to the table. The sample container completely covers the coil for maximum field interaction. Because the thickness of the samples is smaller than the separation distance, the distance between the coils is maintained at 40 mm for all tests (see Supplementary Table S1). All experimental setups for this study are conducted in a standard laboratory environment. Details for specific methods are described below.

#### Method to evaluate power transfer efficiency

Wireless power transfer efficiency *η* (dimensionless) is defined as the ratio of direct current (DC) power delivered to a load by the receiver *P*_*L*_ (W) to transmitted wireless power from the transmitter *P*_*TX*_ (W) as described by Eq. (6).

6 $$\eta =\frac{{P}_{L}}{{P}_{TX}}$$

Power transfer efficiency is an end-to-end system performance parameter and is a primary indicator of the charging system’s functionality. Understanding how the efficiency varies in the presence of lunar simulants is an important consideration for lunar surface wireless charging applications. The WiBotic web-based system control panel is used to measure *P*_*TX*_ and *P*_*L*_. Average steady-state values are used to calculate efficiency per Eq. (6).

#### Method to measure power dissipation

Thermal response is monitored as an additional indicator of power dissipation. Understanding heating mechanisms and thermal effects that result from incident field interaction with the regolith is especially important for the thermal management system of any planned mission to the lunar surface. Measurements are made using the same commercial WiBotic wireless power transfer system and set up described above. To measure temperature, a FLIR EX6 thermal imaging camera is used. Images are captured prior to the start of each test, including a control sample for reference and calibration. For steady-state thermal tests, cycle duration is approximately 1 h.

#### Resonant frequency measurement method

Frequency response data are utilized to assess shifts in the resonant frequency of the transmit and receive coils in the presence of samples. Measurements are made using a calibrated vector network analyzer (VNA), specifically a handheld NanoVNA-F by Deepelec. NanoVNASaver software is used to capture, store, and analyze the measured scattering parameter data, including return loss (S_11_) for the desired frequency range with N = 101 samples between 6.1 and 7.5 MHz. Baseline measurements are made with no samples present. Following this, measurements are made for each coil with one sample at a time placed on top of the face of the coil under test. Samples are centered, on-axis, and adjacent to the coil under test.

## Results

In this section, experimental results are presented for the pursued investigation described above, including power transfer efficiency, power dissipation (heating), and resonant frequency shift when lunar simulants with varied content of metallic iron are introduced at the surface of the power transfer coils. Discussion follows with specific focus on the role of particle size, PSSDR, and their impact to attenuation.

### Wireless power transfer with magnetically coupled resonators in presence of lunar simulants

**Figure 3:**
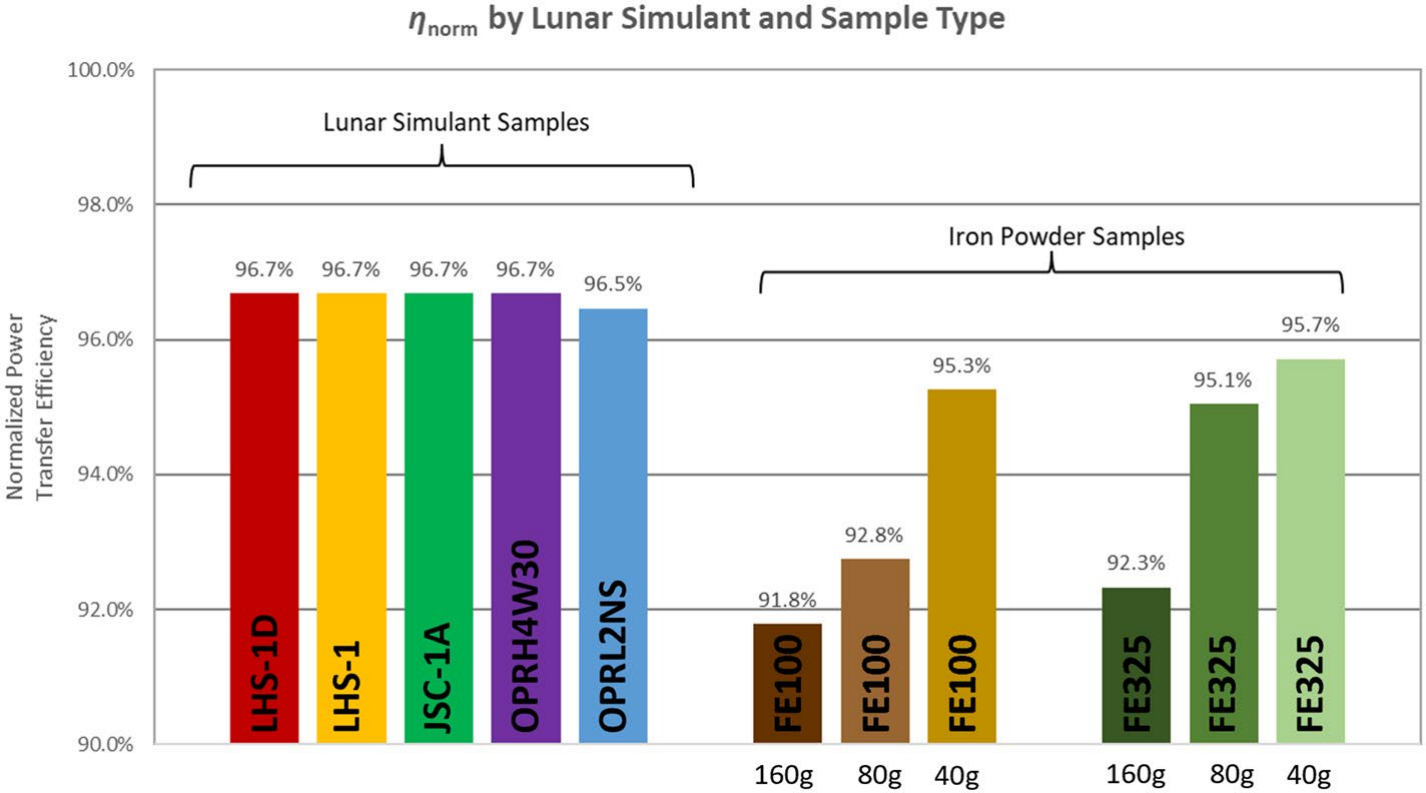
Measured power transfer efficiency is shown for five lunar simulant samples plus six iron powder samples; results have been normalized to the peak $\eta$ with no simulant present by dividing measured efficiency for each case by the peak $\eta$ with no simulant present. For each test, separation between TC and RC is ~ 40 mm with sample between coils. Results show negligible effect by presence of standard lunar simulants but presence of iron-enriched simulant and iron powders cause increasingly degraded efficiency, particularly as iron powder surface density increases. Additionally, 150 μm particle size impacts efficiency more than 45 μm particle size.

This section presents results for power transfer efficiency in the presence of various standard lunar simulants, iron powders, and the new iron-enriched simulant OPRL2NS. Figure 3 shows measured $\eta$ for five lunar simulant samples plus six iron powder samples. *P*_*TX*_ and *P*_*L*_ are measured, and efficiency is calculated per Eq. (6). Efficiency values in the figure have been normalized to the maximum efficiency achieved with no simulant present, specifically by taking the ratio of the measured efficiency with each sample to the maximum measured efficiency with no simulant. Results show that the standard lunar simulants tested have negligible effect on $\eta$ when they are present between the coils, while the iron-enriched simulant and iron powders are shown to reduce the power transfer efficiency. Furthermore, it is observed that efficiency degrades as iron particle size increases; 150 μm iron powder impacts efficiency more than 45 μm iron powder. Efficiency also degrades as iron powder surface density increases.

### Power dissipation and thermal response in presence of lunar simulants

**Figure 4:**
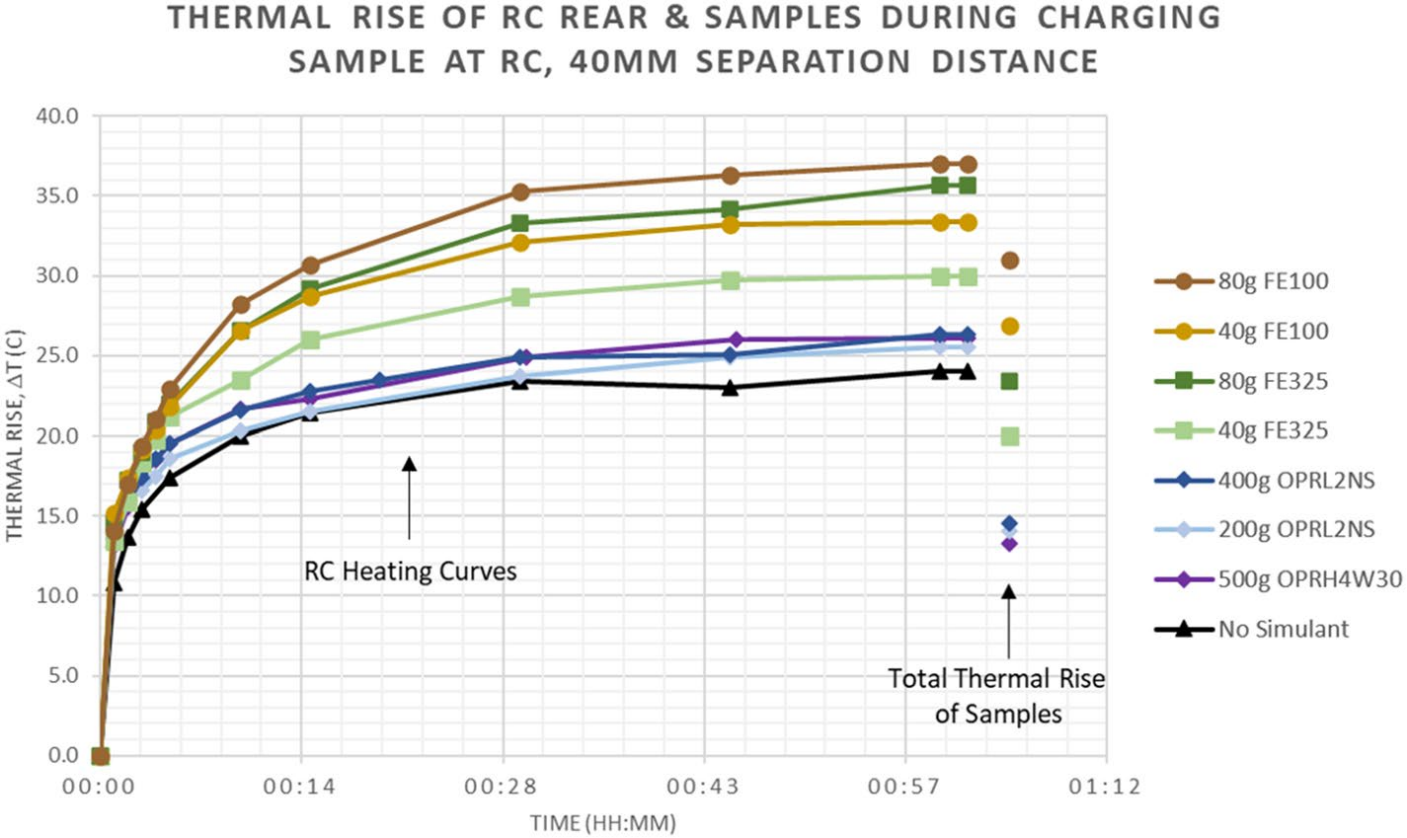
Thermal rise is shown for receive coil (RC) over duration of 1 h (curves) as well as for samples of lunar simulants and iron powders (stand-alone symbols after 1 h). Results show negligible effect by presence of lunar simulants. However, iron powders cause increased heating of RC, and heating increases with surface density of sample. Of all samples, 80 g FE100 sample heats the most (ΔT =  + 31.0° after 1 h). Of the samples with 40 g metallic iron, heating follows particle size with the 25 nm iron-enriched simulant heating the least (ΔT =  + 14.5°) and the 150 μm iron heating the most (ΔT =  + 26.9°). Thermal rise of sample is not measured for the No Simulant case because there is no sample present.

In this section, experimental results are presented for thermal rise of the resonant coils and simulants themselves due to the presence of lunar simulants and metallic iron powders in the wireless power transfer field. Figure 4 shows the measured thermal rise for the rear side of the receiving coil (RC) during normal charging for a duration of 1 h, as well as the measured thermal rise for a subset of samples of lunar simulants and iron powders. The RC is observable during the course of the charging cycle, hence data is shown as thermal response curves over time. However, the samples are only observable before and after the charging cycle, so only the total thermal rise is shown; these data are represented by the stand-alone symbols to the right of the RC thermal response curves. Results show that the presence of lunar simulants increases the receive coil temperature by an additional 1°–2° over the baseline case with no simulant present. The simulants themselves experience 13°–14° of heating, and the iron-enriched simulant heats 1°–2° more than the standard lunar simulant. Iron powders cause significantly more heating of the receive coil, particularly as the surface density of samples increases. Specifically, presence of the highest density sample of the largest grain size (80 g FE100) causes the greatest heating of the coil, at ΔT_RC_ of + 37.0°. Less coil heating is observed in the presence of the super-fine FE325 samples. Considering the thermal rise of the samples themselves, the 80 g FE100 sample experiences the largest temperature increase, ΔT_sample_ of + 31.0° compared to + 23.4° for the equivalent amount of FE325. Of the samples with equivalent surface densities of metallic iron (40 g FE325, 40 g FE100, and 400 g OPRL2NS), the total thermal rise after 1 h follows particle size, with the 25 nm iron-enriched simulant heating the least (ΔT =  + 14.5°), the 45 μm iron powder heating more (ΔT =  + 20.0°), and the 150 μm iron heating the most (ΔT =  + 26.9°).

### Resonant frequency of magnetically coupled resonators in presence of lunar simulants

**Figure 5:**
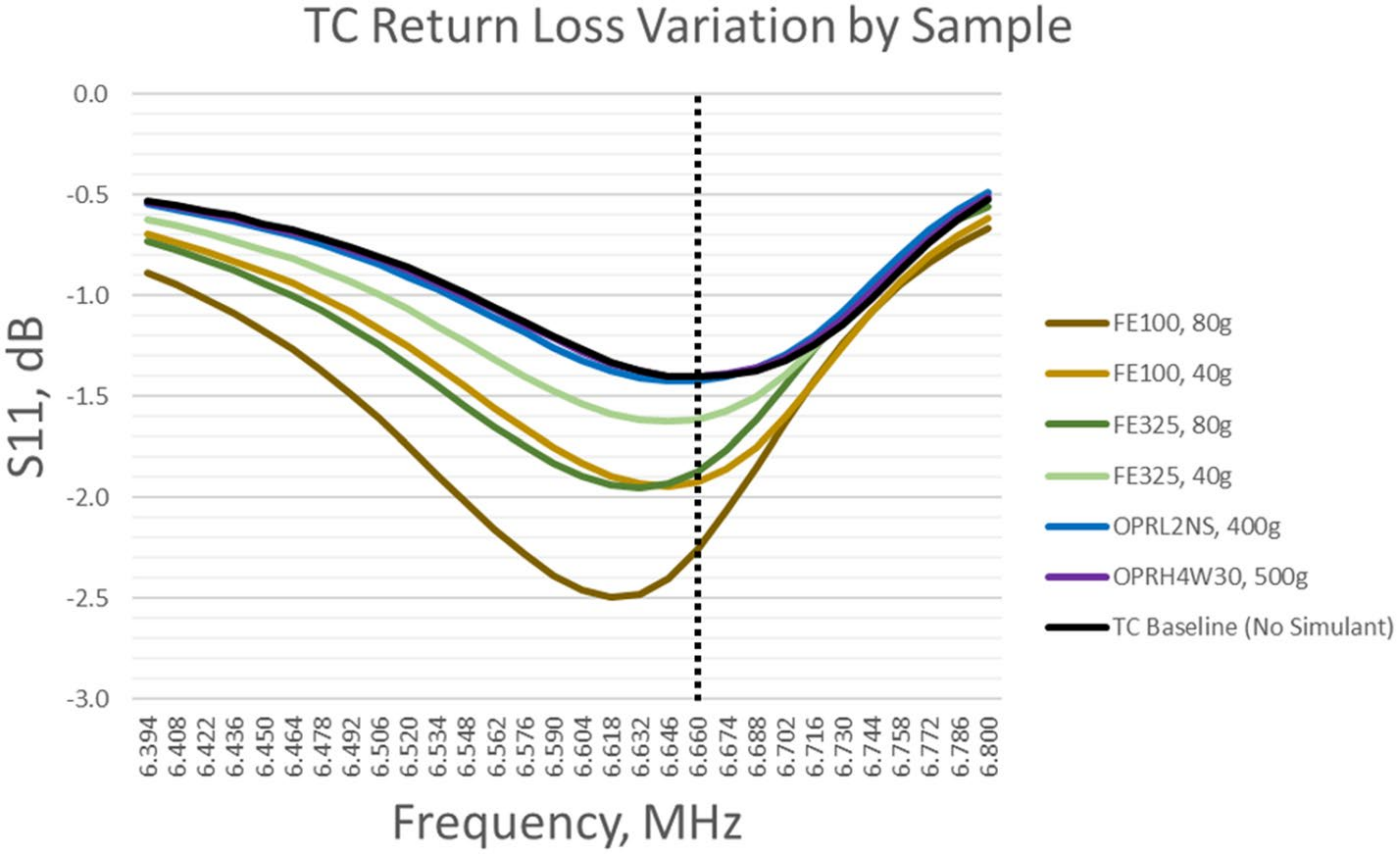
Return loss (S_11_) is shown for transmit coil (TC) with no simulant, with samples of lunar simulants, and with iron powders. Resonant frequency is indicated by S_11_ minima, and the baseline *f*_*0*_ is indicated by the dashed line. Results show presence of standard lunar simulant has no measurable effect on resonant frequency, as evidenced by the overlap of the OPRH4W30 curve with the baseline curve. However, iron-enriched simulant detunes by −14 kHz (0.2%). Presence of iron powders is shown to increase detuning, particularly as surface density increases; detuning is more pronounced with 150 μm particle sizes than 45 μm particle sizes.

This section presents results for resonant frequency of the power transfer coils in the presence of various standard lunar simulants, iron powders, and iron-enriched simulant OPRL2NS. Figure 5 shows measured return loss for the transmit coil under various conditions. Return loss, also known as scattering parameter S_11_ (dB), is commonly used to evaluate antenna resonant frequency, indicated by the frequency response minima. The transmit coil used in this study is shown to have a resonant frequency of 6.660 MHz with no simulant present (black curve). In the presence of standard lunar simulant OPRH4W30, the coil is found to have an identical response, and no detuning is observed (purple curve). However, iron-enriched simulant OPRL2NS (blue curve) is found to shift the resonant frequency by −14 kHz (0.2%). The largest amount of frequency shift occurs in presence of the iron powders (green curves and brown curves), and detuning is shown to increase with surface density for a given particle size. Furthermore, different particle sizes of iron powders are found to introduce different degrees of detuning; presence of the 150 μm particles shifts the resonance by −42 kHz (0.6%) compared to −14 kHz (0.2%) in presence of the 45 μm particles.

## Discussion

The measured results reported for standard lunar simulants demonstrate minimal interaction between non-magnetic samples and a high frequency magnetic field, as expected. On the other hand, results for iron-enriched lunar simulant and pure iron powder samples reveal important insights regarding the relationship between particle size and magnetic field coupling. Furthermore, results show that lunar simulant samples containing iron in the form of iron oxide (Fe_x_O_y_) show no increased coupling; however, presence of metallic iron (Fe^0^) is a determining factor. The low magnetic permeability of iron oxides compared to metallic iron explains why the lunar simulants with iron oxides and no metallic iron do not interact with the incident magnetic field in this study, yet the iron-enriched lunar simulant OPRL2NS does. However, the observed coupling of OPRL2NS with the field is much lower than that of the pure iron powder samples. Particle size of the metallic iron is found to be a critical parameter for the observed coupling, as explained below.

In this study, the three samples containing metallic iron are all in the $PSSDR\ll 1$ category based on the estimated attenuation constants for iron powders shown in Table 2, using Eqs. (3a, 3b). Table 2 shows that reported constitutive parameter values for iron powders result in lower attenuation constant values for iron powder compared to solid iron^26–28^. This results in larger skin depth values for iron powders compared to iron in bulk form. For example, with the parameters from Table 2, skin depth for bulk iron is calculated to be approximately ${\delta }_{S}\approx$ 137 nm at 6.78 MHz, while that of iron powders is on the order of millimeter to centimeter values at the reported microwave frequencies (2–30 GHz). While an individual particle with a very low PSSDR will cause a very small amount of field attenuation, it is important to consider the effect of the total volume of particles when comparing across samples. For example, consider the sizes of metallic iron particles in three samples used in this study: (a) FE100, *d* = 150 $\upmu$m, (b) FE325, *d* = 45 $\upmu$m, and (c) OPRL2NS, *d* = 25 nm iron particles. Although the attenuation of an incident field due to each 25 nm particle will be much smaller than the attenuation due to each 150 $\upmu$m and 45 $\upmu$m particle, the cumulative attenuation due to the volume of each sample must be considered in order to determine the resultant power dissipation for the system. Comparing across samples, the numbers of particles (${N}_{i}$) in the samples are inversely related by their relative particle sizes, because the samples have identical mass and volume of metallic iron. Hence,

**Table 2:** Constitutive parameters and attenuation constant for bulk iron, iron powder, lunar regolith, and simulant samples.

Category	References	Reported						Calculated
		σ (S/m)	ε_r′_	ε_r_″	Μ_R′_	μ_r_″	*f *(Hz)	α (Np/m)
Solid iron (purified)		1.00E+07	1.00E+00	0.00E+00	2.00E+05	0.00E+00	6.78E+06	7.32E+06
Solid iron		1.00E+07	1.00E+00	0.00E+00	5.00E+03	0.00E+00	6.78E+06	1.16E+06
Iron powder	Yang^26^	1.00E−07	6.00E+01	5.00E+00	6.00E+00	5.50E+00	2.00E+09	3.72E+02
Iron powder	Yang^26^	1.00E−07	6.00E+01	7.50E+00	1.00E+00	3.50E+00	1.00E+10	2.02E+03
Iron powder	Yang^26^	1.00E−07	6.00E+01	1.05E+01	1.00E+00	2.00E+00	1.80E+10	2.63E+03
Iron powder	Devi^36^	6.37E−01	7.07E+01	3.82E−01	2.87E+01	3.33E+00	3.00E+10	1.80E+03
Iron powder	Buchelnikov^27^	1.00E−07	1.20E+01	3.00E−01	1.00E+00	1.00E−01	2.45E+09	1.11E+01
Iron powder	Semenenko^28^	1.00E−07	2.00E+01	8.00E−01	3.00E+00	1.75E+00	3.00E+09	1.47E+02
Iron powder	Semenenko^28^	1.00E−07	1.50E+01	4.00E−01	2.80E+00	1.50E+00	3.00E+09	1.11E+02
Iron powder	Semenenko^28^	1.00E−07	1.60E+01	5.00E−01	2.60E+00	1.25E+00	3.00E+09	1.01E+02
Iron powder	Semenenko^28^	1.00E−07	1.20E+01	3.00E−01	2.40E+00	1.00E+00	3.00E+09	7.32E+01
Regolith	Lai^29^	2.00E−03	3.50E+00	8.02E−02	1.00E+00	0.00E+00	6.00E+07	2.27E−01
Regolith	Lai^29^	2.00E−03	3.50E+00	3.68E−02	1.00E+00	0.00E+00	6.00E+07	2.13E−01
Regolith	Wang^30^	2.00E−03	2.90E+00	1.45E−02	1.00E+00	0.00E+00	6.00E+07	2.25E−01
Regolith	Lai^31^	2.00E−03	3.50E+00	4.55E−01	1.00E+00	0.00E+00	6.00E+07	3.50E−01
Regolith	Barmatz^32^	1.00E−14	3.73E+00	4.70E−02	1.00E+00	2.00E−03	2.00E+09	5.91E−01
Regolith	Barmatz^33^	1.00E−14	3.73E+00	4.40E−02	1.03E+00	5.60E−03	3.50E+09	1.24E+00
Mars simulant	Stillman^17^	6.67E−05	1.80E+00	0.00E+00	1.00E+00	0.00E+00	1.00E+07	9.35E−03
Mars simulant	Stillman^17^	6.67E−05	5.30E+00	0.00E+00	1.00E+00	0.00E+00	1.00E+07	5.45E−03
Iron oxides	Stillman^17^	6.67E−05	1.06E+01	0.00E+00	4.89E+00	0.00E+00	1.00E+07	8.53E−03
Iron oxides	Stillman^17^	6.67E−05	6.92E+00	0.00E+00	2.35E+00	0.00E+00	1.00E+07	7.32E−03
Iron oxides	Stillman^17^	6.67E−05	4.50E+00	0.00E+00	1.16E+00	0.00E+00	1.00E+07	6.38E−03
Iron oxides	Stillman^17^	6.67E−05	3.10E+00	0.00E+00	1.00E+00	0.00E+00	1.00E+07	7.13E−03
Simulant	Martella^34^	6.67E−05	2.20E+00	1.00E−02	1.00E+00	0.00E+00	1.00E+07	9.17E−03
Simulant	Martella^34^	6.67E−05	2.90E+00	2.00E−02	1.00E+00	0.00E+00	1.00E+07	8.60E−03
Simulant	Allan^35^	6.67E−05	5.00E+00	1.00E−01	1.00E+00	0.00E+00	2.45E+09	1.15E+00

7 $$\frac{{N}_{a}}{{N}_{b}} ={\left(\frac{{d}_{b}}{{d}_{a}}\right)}^{3},~~   \frac{{N}_{b}}{{N}_{c}} ={\left(\frac{{d}_{c}}{{d}_{b}}\right)}^{3} ~\mathrm{and  }~~\frac{{N}_{c}}{{N}_{a}} ={\left(\frac{{d}_{a}}{{d}_{c}}\right)}^{3}.$$

From Eq. (7), it can be concluded that the number of 25 nm particles in the OPRL2NS sample is 2.16 $\times$ 10^11^ times greater than the number of 150 $\upmu$m particles in the FE100 sample and 5.83 $\times$ 10^9^ times greater than the number of 45 $\upmu$m particles in the FE325 sample. However, this cubic increase in number of particles is outweighed by a quintic decrease in absorbed power per particle as shown in Eq. (5). Specifically, taking the total absorbed power for a sample as the product of ${P}_{i}$ and ${N}_{i}$, and incorporating Eqs. (5) and (7), total absorbed power for two samples can be compared as *ρ*, the ratio of total power absorbed in sample *a* to total power absorbed in sample *b*:

8 $$\rho = \frac{{P}_{a}{N}_{a}}{{P}_{b}{N}_{b}} = \frac{\kappa {d}_{a}^{3}{\left(\frac{{d}_{a}}{{\delta }_{S}}\right)}^{2}}{\kappa {d}_{b}^{3}{\left(\frac{{d}_{b}}{{\delta }_{S}}\right)}^{2}} \times {\left(\frac{{d}_{b}}{{d}_{a}}\right)}^{3} = {\left(\frac{{d}_{a}}{{d}_{b}}\right)}^{2}.$$

Equation (8) shows that for the same volume of metallic iron, samples with smaller particle size yield the smallest attenuation, and samples with larger particle size yield the largest attenuation, for cases when $PSSDR\ll 1$. For example, comparing the FE100 and FE325 samples using Eq. (8) suggests the total power absorbed in the FE100 sample should be greater than that in the FE325 sample by a factor of ${\left(150\mathrm{ \upmu m}/45\mathrm{ \upmu m}\right)}^{2}\approx$ 11. This is consistent with the measured wireless power transfer efficiency and thermal response results presented in Figs. 3 and 4, respectively. Specifically, the degraded efficiency of the OPRL2NS, FE325, and FE100 samples follows particle size. FE100’s larger particle size implies this sample couples better with the incident field, leading to degraded power transfer efficiency and increased power dissipation (heat), which is consistent with the measured results. Moreover, the OPRL2NS shows the lowest amount of coupling across these three sample groups, which makes sense given this sample contains the smallest particle size of metallic iron.

**Figure 6:**
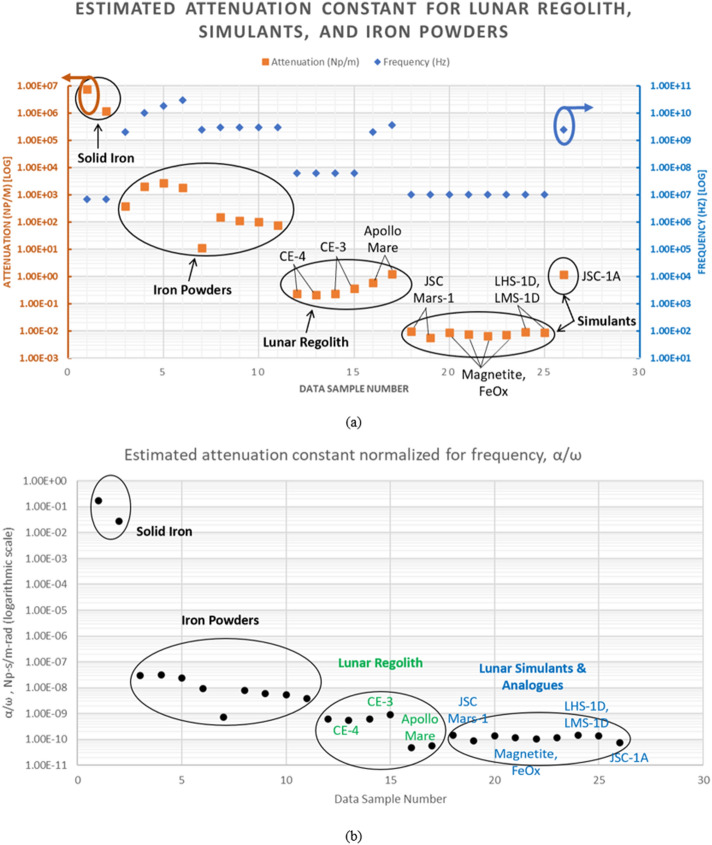
Plots of estimated attenuation constant, α (Np/m), for the reported samples from Table 2. (**a**) Attenuation constant and frequency are plotted for each sample; note both y-axes are on a logarithmic scale. General trends in attenuation constant can be observed, with like materials exhibiting similar values, as indicated by ellipsoids. The dependence on frequency is also apparent, and it can be seen that attenuation constants increase or decrease following the frequency. (**b**) The attenuation constant has been normalized by dividing by frequency, showing trends across sample groups are more consistent.

Table 2 also includes reported constitutive parameter values for other particulate samples, including experimental measurements for actual lunar regolith and simulants at various frequencies between 1 MHz and 30 GHz^17, 29–36^. Figure 6 shows a plot of the estimated attenuation constant, $\alpha$ (Np/m), for the reported samples. The well-known relationship between attenuation and frequency is highlighted in Fig. 6a where some amount of consistency can be seen among the groupings of similar sample types, e.g., iron powders, lunar regolith, and lunar simulants, as indicated by the ellipsoids. In Fig. 6b, attenuation constant has been normalized for frequency. Specific regolith and simulant samples are labeled, including recently reported data from the Chinese lunar rover mission at sites Chang’E-3 (CE-3) and Chang’E-4 (CE-4)^29–31^. Measured data are also shown for Apollo Mare regolith samples^32, 33^, Johnson Space Center Mars-1 simulant (JSC Mars-1)^17^, Lunar Highlands simulant (LHS-1D) and Lunar Mare simulant (LMS-1D)^34^, and Johnson Space Center Lunar Mare simulant (JSC-1A)^35^. Normalizing the calculated attenuation constant values for these various materials highlights the consistency of the trends across sample groups. Results of this study are directly applicable to engineers and scientists working with electromagnetic fields at interfaces with particulate media, including ground penetrating radar (GPR), wireless networks such as cellular/LTE, WLAN, LoRa, IoT, and microwave/mm-wave power beaming networks. Results are particularly relevant for soils with mineralogical composition including conductive and/or magnetic particulate materials, such as metallic iron.

## Conclusions and future work

This work demonstrates the underlying physical principles which govern the exchange of energy between an incident electromagnetic field and particulate materials, and presents specific insights regarding the coupling of a magnetic wireless power transfer field with lunar regolith and simulants with magnetic content. How wireless power transfer efficiency, thermal response, and frequency response are affected by particle size, particularly as particle size relates to the frequency-dependent skin depth parameter, holds important implications for future work involving electromagnetic field interactions with lunar regolith, Martian regolith, and their simulants. For example, while results of this study suggest MCR-based wireless charging should be feasible for upcoming lunar missions, our findings also suggest that additional data is required in order to develop accurate predictions of electromagnetic coupling in the presence of lunar regolith. Specifically, measurements of lunar regolith’s complex permittivity, permeability, and conductivity at wireless charging frequencies would improve coupling predictions which would, in turn, support more accurate mission planning, e.g., estimating how long it will take a lunar rover to charge its battery. Furthermore, the development of simulants which more accurately represent the measured EM properties of regolith at the target frequencies would aid in the experimental validation of the improved predictive models. Results are also relevant to terrestrial projects in which electromagnetic fields interface with particulate media.

While the results of this study are consistent with the governing principles of EM coupling with magnetic material, there are additional considerations which may warrant further research. For example, for magnetic materials in particular, the behavior of magnetic nanoparticles may contribute to these results. Specifically, research has shown that ferromagnetic particles exhibit different behavior when particles become small enough to be considered single domain (all magnetic dipoles within the particle are oriented in the same direction, and no domain walls exist) versus particles with multiple magnetic domains. Various ferromagnetic particles are known to behave as single domain particles at sizes smaller than 100 nm and behave as superparamagnetic as sizes decrease to 30 nm and below^37, 38^. This phenomenon may also help explain the limited coupling observed with the 25 nm iron particles in the OPRL2NS sample.

Particle shape, specifically sphericity, is another important consideration. SEM images of the samples show that the FE325 particles are mostly spherical, while the FE100 samples include irregular shapes (see Fig. 2e,f). It has been demonstrated that elongated and flattened particle shapes lead to increased electrical conductivity in particulate matter^39^. Increased electrical conductivity in the FE100 sample could contribute to increased attenuation in the sample, through the eddy current generated by the induced field, as mentioned previously. Hence, particle sphericity is another important parameter to consider when evaluating EM fields at interfaces with particulate media. Future work could include development of a model incorporating electrical conductivity based on the sphericity of metallic iron particle samples. This would complement the findings presented in this work regarding the relationship between particle size and coupling with the magnetic field.

A third area to note is the frequency and temperature dependence of the constitutive parameters reported in the literature for lunar regolith and simulants. It is well-established that permittivity and permeability vary over frequency and temperature, yet scant data exists in the literature below microwave frequencies or at lunar surface temperatures. As discussed previously, accurate estimation of attenuation constant depends on these values. Future research in this area would benefit the broader space research community.

## Supplementary Information

Supplementary Information.

## Data Availability

All data generated or analyzed during this study are included in this published article and its supplementary information files.
