# Supplementary material for: Magnetic field coupling with lunar soil simulants
Source: Sci Rep. 2023 Jun 15;13:9713. doi: 10.1038/s41598-023-36527-0 (PMC10272116; doi:10.1038/s41598-023-36527-0)
Supplement: Supplementary file 1 — Supplementary Information. [file 41598_2023_36527_MOESM1_ESM.pdf]

Supplemental Information for “Magnetic field coupling with Lunar soil simulants”

Supplementary Table S1. Samples of Lunar Simulants and Iron Powders

Sample	Type	Grain Size	Mass	Container	Thickness	Area	Surface Density*	Volume	Volume Density**
#		m	g		mm	cm^2	D_s (g/cm^2)	cm^3	D_v (g/cm^3)
1	LHS-1	<400u	350	Z-S+Q	15	245.85	1.42	368.78	0.95
2	Fe100	<100u	350	Z-S+Q	12	245.85	1.42	295.02	1.19
3	LHS-1	<400u	1000	Z-G	15	731.64	1.37	1097.46	0.91
4	LHS-1D	<35u	1000	Z-G	15	731.64	1.37	1097.46	0.91
5	Fe100	<100u	160	Z-G	2	731.64	0.22	146.33	1.09
6	Fe325	<45u	160	Z-G	2	731.64	0.22	146.33	1.09
7	Fe325	<45u	350	Z-S+Q	12	245.85	1.42	295.02	1.19
8	OPRH4W30	unknown	500	Z-S+Q	20	245.85	2.03	491.70	1.02
8B	OPRH4W30	unknown	500	Z-2G	2	1257.30	0.40	251.46	1.99
9	OPRH4W30	unknown	100	OPR	n/a				
10	JSC-1A	unknown	350	Z-S+Q	14	245.85	1.42	344.19	1.02
11	Light Dusting of Fe100	<100u	80	Z-G	1	731.64	0.11	73.16	1.09
12	Light Dusting of Fe325	<45u	80	Z-G	1	731.64	0.11	73.16	1.09
13	Lighter Dusting of Fe100	<100u	40	Z-G	1	731.64	0.05	73.16	0.55
14	Lighter Dusting of Fe325	<45u	40	Z-G	1	731.64	0.05	73.16	0.55
15	Lightest Dusting of Fe100	<100u	10	Z-G	1	731.64	0.01	73.16	0.14
16	Lightest Dusting of Fe325	<45u	10	Z-G	1	731.64	0.01	73.16	0.14

KEY	Container	W (cm)	L (cm)	A (cm^2)
Z-S	ziploc, sandwich size	16.5	14.9	245.85
Q	ziploc, quart zipper	20	14.9	298
Z-G	ziploc, gallon	26.8	27.3	731.64
Z-2G	ziploc, 2-gallon	33	38.1	1257.3

*In these measurements, surface density gives a better idea of how much material was coating the coil surface.

**Volume density must be considered in light of thickness and total mass of sample.
